# Rheumatoid arthritis-associated polymorphisms are not protective against Alzheimer's disease

**DOI:** 10.1186/1750-1326-6-33

**Published:** 2011-05-19

**Authors:** Christopher R Simmons, Fanggeng Zou, Steven G Younkin, Steven Estus

**Affiliations:** 1 Department of Physiology, Sanders-Brown Center on Aging, University of Kentucky, Lexington, KY, USA; 2 Department of Neuroscience, Mayo Clinic College of Medicine, Jacksonville, FL, USA

## Abstract

**Background:**

Rheumatoid arthritis (RA) and Alzheimer's disease (AD) are inversely associated. To test the hypothesis that genetic elements associated with increased RA risk are associated with decreased AD risk, we evaluated RA genetic risk factors recently identified in genome-wide association studies (GWAS) for their association with AD in a two-stage, case-control analysis.

**Results:**

In our Stage 1 analysis of ~800 AD and ~1,200 non-AD individuals, three of seventeen RA-associated SNPs were nominally associated with AD (p < 0.05) with one SNP, rs2837960, retaining significance after correction for multiple testing (p = 0.03). The rs2837960_G (minor) allele, which is associated with increased RA risk, was associated with increased AD risk. Analysis of these three SNPs in a Stage 2 population, consisting of ~1,100 AD and ~2,600 non-AD individuals, did not confirm their association with AD. Analysis of Stage 1 and 2 combined suggested that rs2837960 shows a trend for association with AD. When the Stage 2 population was age-matched for the Stage 1 population, rs2837960 exhibited a non-significant trend with AD. Combined analysis of Stage 1 and the age-matched Stage 2 subset showed a significant association of rs2837960 with AD (p = 0.002, OR 1.24) that retained significance following correction for age, sex and APOE (p = 0.02, OR = 1.20). Rs2837960 is near *BACE2*, which encodes an aspartic protease capable of processing the AD-associated amyloid precursor protein. Testing for an association between rs2837960 and the expression of *BACE2 *isoforms in human brain, we observed a trend between rs2837960 and the total expression of *BACE2 *and the expression of a *BACE2 *transcript lacking exon 7 (p = 0.07 and 0.10, respectively).

**Conclusions:**

RA-associated SNPs are generally not associated with AD. Moreover, rs2837960_G is associated with increased risk of both RA and, in individuals less than 80 years of age, with AD. Overall, these results contest the hypothesis that genetic variants associated with RA confer protection against AD. Further investigation of rs2837960 is necessary to elucidate the mechanism by which rs2837960 contributes to both AD and RA risk, likely via modulation of *BACE2 *expression.

## Background

There is a long-standing, inverse relationship between the prevalence of Alzheimer's disease (AD) and of rheumatoid arthritis (RA). Jenkinson and colleagues first described the decreased prevalence of RA in patients suffering from senile dementia of the Alzheimer's type as compared to cognitively intact individuals [1]. Further retrospective studies of clinical and autopsy data revealed that patients with RA exhibit a reduced prevalence of AD [2]. A study by Myllykangas-Luosujarvi and colleagues evaluating AD pathology in patients with and without RA revealed that AD-associated neuropathology occurred four times less often in patients with RA as compared to the general population [3].

The basis of this inverse relationship is unclear but may include both genetic and environmental factors. RA and AD each have a strong genetic component, i.e., 50% of RA risk and 60% of AD risk is attributable to genetic factors, supporting the original hypothesis of Jenkinson and colleagues that genetics might explain the relationship between AD and RA [4,5]. Alternatively, anti-inflammatory medications used therapeutically for the treatment of RA could decrease AD risk by reducing AD-associated inflammation or via other mechanisms, .e.g., modulation of APP processing [6,7]. Supporting this possibility, an initial double-blind, placebo-controlled study by Rogers *et al. *provided evidence that indomethacin slowed cognitive decline in patients with AD relative to placebo [8]. These findings were further supported by Breitner and colleagues who found that multiple anti-inflammatory medications slow disease progression and delay disease onset [9]. However, there has been little success replicating these findings in larger, randomized clinical trials [10-13]. Hence, whether anti-inflammatory agents delay the onset of AD remains unclear.

The recent advent of RA genome wide association studies (GWAS) has identified single nucleotide polymorphisms (SNP)s associated with RA that provide a foundation for evaluating the initial hypothesis of Jenkinson *et al. *that genetic variants that increase the risk of RA also decrease the risk of AD. To this end, we tested whether seventeen RA-associated SNPs with genome-wide significance were associated with AD in a two-stage analysis using separate AD case-control populations. We found that none of the seventeen alleles associated with increased RA risk were also associated with reduced AD risk. Rather, we found three RA-associated SNPs that were nominally associated with AD (p < 0.05). One of these SNPs, rs2837960, was found to be significantly associated with AD in a combined analysis of our Stage 1 and Stage 2 populations when the Stage 2 population was restricted to individuals of similar age as Stage 1. The gene closest to rs2837960 is *BACE2*, the product of which has been implicated in amyloid protein precursor (APP) processing [14,15]. When we evaluated the expression of *BACE2 *isoforms as a function of rs2837960, we found a trend for *BACE2 *expression with rs2837960. In summary, genetic variants that increase RA risk do not decrease AD risk. The inverse relationship between RA and AD may thus be better explained by environmental factors such as the use of anti-inflammatory medications. Further functional investigation of rs2837960 is needed to elucidate the mechanism by which this SNP may modulate AD and RA.

## Results

### RA-associated SNPs are generally not associated with AD

To evaluate whether RA-associated SNPs are also associated with AD, we began by identifying SNPs that are robustly associated with RA risk and then evaluated these SNPs for their association with AD in an exploratory Stage 1 case-control population of ~800 AD and ~1200 non-AD individuals. Contrary to the hypothesis that alleles associated with increased RA risk are also associated with reduced AD risk, only three of the seventeen RA-associated SNPs in our Stage 1 study were nominally significant for association with AD (p < 0.05, Table 1). Moreover, for each these SNPs, the allele associated with increased RA risk was also associated with increased AD risk, further refuting the hypothesis that genetics underlies the inverse epidemiologic relationship between RA and AD prevalence. The AD-associated SNPs are located in or near the genes *BACE2 *(rs2837960; p = 0.002, OR = 1.29), *TRAF1/C5 *(rs3761847; p = 0.006, OR = 1.19) and *SALL3 *(rs2002842; p = 0.04, OR = 1.15). When a Bonferroni correction for multiple testing was applied to minimize false-positive associations between RA-associated SNPs and AD, only rs2837960 exhibited a significant association with AD (p = 0.03). Furthermore, rs2837960 remained significantly associated with AD after correcting for age, sex and *APOE *genotype per logistic regression (p = 0.012 OR = 1.26, Table 2). Rs3761847 remained significant following correction of Stage 1 analysis for covariates (p = 0.007 OR = 1.21).

**Table 1:** Stage 1 Analysis of RA-SNPs Association with AD

CHR	SNP	Gene	RA OR	RA P-value	AD OR [96% CI]	AD P-value	BF p-value
**21**	rs2837960	BACE2	1.05	2 × 10^-6^	1.29 [1.10-1.52]	*0.002*	*0.03*
**9**	rs3761847	TRAF1, C5	1.32	4 × 10^-14^	1.19 [1.05-1.35]	*0.006*	0.11
**18**	rs2002842	SALL3	1.61	6 × 10^-6^	1.15 [1.01-1.31]	*0.04*	0.65
**9**	rs881375	TRAF1, C5	NR	4 × 10^-8^	1.11 [0.97-1.26]	0.12	
**6**	rs660895	HLA-DRB1	3.62	1 × 10^-108^	0.93 [0.80-1.09]	0.39	
**12**	rs3184504	SH2B3	0.92	6 × 10^-6^	0.95 [0.84-1.08]	0.41	
**8**	rs2736340	BLK	1.19	6 × 10^-9^	0.95 [0.82-1.10]	0.47	
**2**	rs13031237	REL	1.13	8 × 10^-7^	1.04 [0.92-1.19]	0.52	
**6**	rs6457617	HLA-E	2.36	5 × 10^-75^	1.03 [0.91-1.17]	0.61	
**4**	rs13119723	IL2, IL21	1.12	7 × 10^-7^	0.96 [0.80-1.14]	0.62	
**2**	rs13017599	REL	1.21	2 × 10^-12^	1.03 [0.90-1.17]	0.67	
**9**	rs951005	CCL21	0.81	4 × 10^-10^	1.03 [0.87-1.22]	0.73	
**6**	rs6910071	HLA-DRB1	2.88	1 × 10^-299^	0.98 [0.84-1.14]	0.75	
**1**	rs2476601	PTPN22	1.94	9 × 10^-74^	1.03 [0.84-1.27]	0.77	
**7**	rs10488631	IRF5	1.19	4 × 10^-11^	0.99 [0.81-1.20]	0.91	
**2**	rs231735	CTLA4	0.83	6 × 10^-9^	1.01 [0.89-1.14]	0.91	
**1**	rs3890745	TNFRSF14	0.88	4 × 10^-6^	0.99 [0.87-1.13]	0.93	

Seventeen SNPs exhibiting genome-wide significant associations with RA were tested for their association with AD risk using allelic models in a Mayo Clinic AD GWAS series of 843 AD and 1264 non-AD individuals. Three of the seventeen RA-associated SNPs were nominally associated with AD (p < 0.05). Multiple testing was addressed by applying a Bonferroni (BF) correction for the number of tests performed. The only SNP that retained significance after BF correction was rs2837960 (p = 0.03). NR = value not reported in initial study.

**Table 2:** Stage 1 Analysis of RA-SNP's Association with AD Corrected for Age, Sex and *APOE *Genotype

CHR	SNP	OR	L95	U95	P
**9**	rs3761847	1.211	1.054	1.392	*0.007*
**21**	rs2837960	1.256	1.051	1.502	*0.012*
**18**	rs2002842	1.147	0.995	1.323	0.059
**9**	rs881375	1.125	0.973	1.300	0.111
**6**	rs660895	0.903	0.759	1.076	0.254
**8**	rs2736340	0.920	0.786	1.077	0.298
**2**	rs13031237	1.067	0.926	1.230	0.372
**6**	rs6457617	1.056	0.921	1.211	0.435
**2**	rs13017599	1.055	0.914	1.217	0.467
**9**	rs951005	1.060	0.880	1.278	0.539
**6**	rs6910071	0.949	0.797	1.130	0.558
**2**	rs231735	1.027	0.895	1.178	0.705
**7**	rs10488631	0.961	0.774	1.193	0.717
**4**	rs13119723	0.969	0.800	1.174	0.746
**1**	rs3890745	1.022	0.882	1.186	0.770
**1**	rs2476601	1.018	0.811	1.278	0.880
**12**	rs3184504	0.998	0.869	1.146	0.976

Using logistic regression, SNP-AD associations were corrected for the potential effects of age, sex and APOE genotype (all of which were independent predictors of AD in our Stage 1 analysis). The minor alleles of both rs2837960 and rs3761847 were significantly associated with increased risk of AD (p < 0.05) while the minor allele of rs2002842 exhibited only a trend toward an increased risk of AD.

### Rs2837960 is significantly associated with AD in individuals

The three RA- and potentially AD-associated SNPs from Stage 1 were evaluated further for their association with AD in a Stage 2 case-control series that consisted of 2677 non-AD and 1102 AD subjects. Contrary to our initial results, these SNPs exhibited no association with AD in our Stage 2 population (Table 3). Correction for age, sex and APOE genotype had marginal, non-significant effects on the association between these three SNPs and AD. Our Stage 1 and Stage 2 populations were combined with the intention of clarifying the overall association between these three SNPs and AD. Analysis of this combined population, consisting of 3949 non-AD and 1965 AD subjects, suggested that rs2837960 is significantly associated with AD (p = 0.04, OR = 1.11, Table 4). When these results were corrected for age, sex and *APOE *genotype this association between rs2837960 and AD showed only a trend (p = 0.14).

**Table 3:** Stage 2 Analysis of Top RA-SNPs Associated with AD

		*Uncorrected Logistic Regression*				*Regression Corrected for Age, Sex and APOE*			
CHR	SNP	OR	L95	U95	P	OR	L95	U95	P
**21**	rs2837960	1.011	0.887	1.153	0.865	1.000	0.867	1.154	0.997
**18**	rs2002842	0.992	0.896	1.099	0.880	0.990	0.886	1.106	0.857
**9**	rs3761847	0.921	0.832	1.019	0.112	0.905	0.809	1.012	0.080

No RA-associated SNP that exhibited an association with AD in Stage 1 was found to exhibit an association with AD in our Stage 2 population. This remained true when data were analyzed and corrected for covariates including age, sex and APOE genotype of Stage 2 individuals, who on average were significantly older than Stage 1 individuals (p < 0.001, Students t-test).

**Table 4:** Combined Stage 1 and Stage 2 Analysis of Top RA-SNPs for Association with AD

*Regression Corrected for Age, Sex and APOE*						*Uncorrected Logistic Regression*			
CHR	SNP	OR	L95	U95	P	OR	L95	U95	P
**21**	rs2837960	1.113	1.007	1.230	*0.037*	1.087	0.974	1.212	0.138
**18**	rs2002842	1.031	0.952	1.116	0.460	1.017	0.933	1.110	0.699
**9**	rs3761847	1.011	0.934	1.093	0.793	1.002	0.920	1.091	0.965

Initial analysis of combined Stage 1 and Stage 2 populations revealed that rs2837960 remained overall nominally significant for association with AD per logistic regression (p < 0.05). When this analysis was repeated to include covariate data, rs2837960 showed only a trend for association with AD.

Due to the large discrepancy in average age between our Stage 1 and Stage 2 populations, and the possibility that the impact of genetic risk factors may decline with age, we next evaluated a subset of Stage 2 individuals that had an age of AD diagnosis between 60 and 80 years of age along with non-AD individuals with the same age range [16]. Thus, our age parameters and average population ages for Stage 2 mimicked those of Stage 1. This effort resulted in a sample population that consisted of 186 AD (average age 73) and 912 non-AD individuals (average age of 74). This population showed a trend in the OR of rs2837960 that was consistent with that of the Stage 1 analysis (Table 5), i.e., the minor rs2837960_G allele appeared to impart an increased, although non-significant, risk of AD. The SNPs rs3761847 and rs2002842, that were associated with AD in Stage 1, failed to show an association with AD in Stage 2. We note that there was significant overlap in the 95% confidence intervals between Stage 1 and Stage 2 for all three of these SNPs. Analysis of the combined data from Stage 1 and the Stage 2 subset revealed that only rs2837960 maintained a significant association with AD (p = 0.002, OR = 1.27, Table 6). This association between rs2837960 and AD remained significant following correction for the covariates age, sex and APOE (p = 0.02, OR = 1.20). Thus, when the Stage 1 and Stage 2 populations were matched for age, rs2837960 was significantly associated with AD risk.

**Table 5:** Analysis of RA-SNP's Association with AD in Stage 2, Age-Matched to Stage 1.

*Uncorrected Logistic Regression*						*Regression Corrected for Age, Sex and APOE*			
CHR	SNP	OR	L95	U95	P	OR	L95	U95	P
**21**	rs2837960	1.218	0.922	1.609	0.165	1.225	0.903	1.664	0.192
**18**	rs2002842	1.002	0.802	1.253	0.983	1.013	0.794	1.292	0.920
**9**	rs3761847	0.897	0.723	1.113	0.324	0.878	0.694	1.110	0.278

The three RA-associated SNPs suggesting association with AD were tested further for their association with AD risk by using logistic in a series of 912 non-AD and 186 AD individuals whose ages more closely resembled those of Stage 1 individuals. Although significant associations were not observed between these SNPs and AD, the results for each SNP were not significantly different from those obtained in Stage 1.

**Table 6:** Analysis of Top RA-SNPs for Association with AD in Combined Stage 1 and Age-Matched Stage 2.

*Uncorrected Logistic Regression*						*Regression Corrected for Age, Sex and APOE*			
CHR	SNP	OR	L95	U95	P	OR	L95	U95	P
**21**	rs2837960	1.239	1.083	1.418	*0.002*	1.199	1.032	1.392	*0.018*
**18**	rs2002842	1.056	0.948	1.178	0.323	1.050	0.931	1.184	0.430
**9**	rs3761847	1.090	0.981	1.210	0.108	1.075	0.958	1.207	0.217

Combined analysis of Stage 1 and Stage 2 data from individuals between 60 and 80 years of age was performed to clarify the association of rs2837960, rs3761847 and rs2002842 with AD risk. The only SNP found to retain a significant association with AD was rs2837960, the G allele of which appears to increase AD risk.

### In silico analyses suggest that rs2837960 is associated with BACE2 expression and splicing

**Figure 1:**
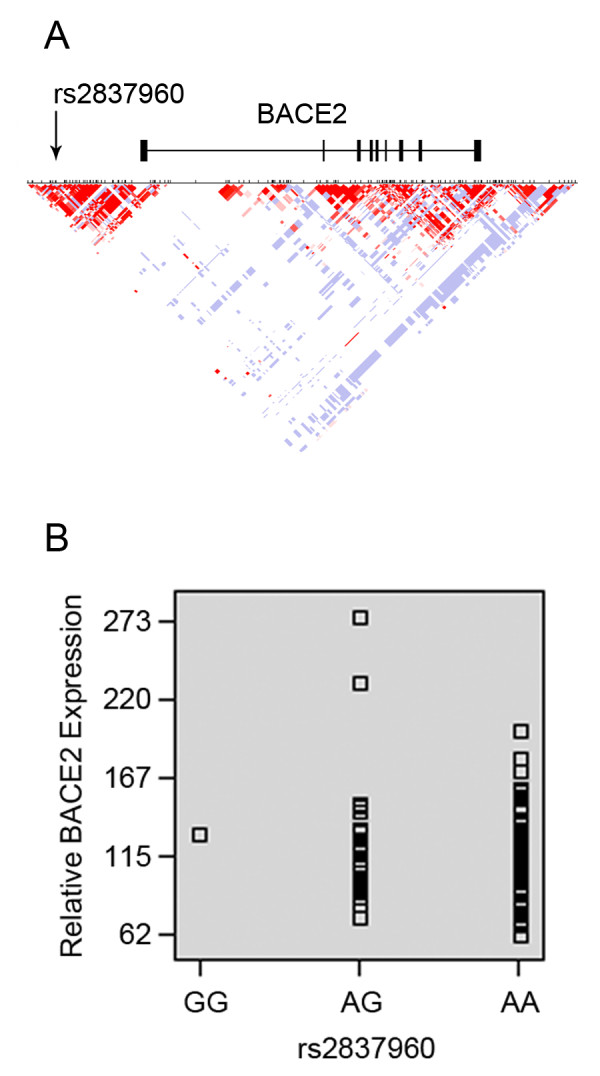
**Location of s2837960 and trend between rs2837960 and *BACE2 *expression**. The gene nearest to rs2837960 is *BACE2*, whose transcription start site is ~27.8 kb downstream. (A) Analysis of the HapMap CEU population reveals that rs2837960 resides within a linkage disequilibrium block that includes the *BACE2 *promoter region and first exon. (B) Analysis of exon tiling array data within the SNPExpress database suggests a trend between rs3837960_G and increased *BACE2 *expression (p = 0.08).

To gain insights into the possible actions of rs2837960, we first identified genes within the vicinity of rs2837960 by using HapMap [17]. This analysis found that rs2837960 resides within a haplotypic block that includes the promoter region and 5'-UTR of *BACE2 *(Figure 1A). Due to the presence of distinct proximal and distal *BACE2 *promoters, and the potential contribution of upstream regulatory elements to *BACE2 *expression, we considered the possibility that rs3846662 may affect *BACE2 *expression [18-20]. We then queried whether there was an association between rs2837960 and *BACE2 *expression in human brain by using the SNPexpress database, which includes data from 93 human brain samples [21]. This analysis revealed a trend towards increased *BACE2 *expression in rs2837960 minor allele carriers (p = 0.08, Figure 1B). Since these data suggest that rs2837960 may modulate *BACE2 *expression and since BACE2 function in turn may modulate AD and cognition, we hypothesized that rs2837960 modulates AD risk by altering *BACE2 *expression in the human brain [22-24].

### Relationship between rs2837960 and BACE2 isoforms in the human brain

**Figure 2:**
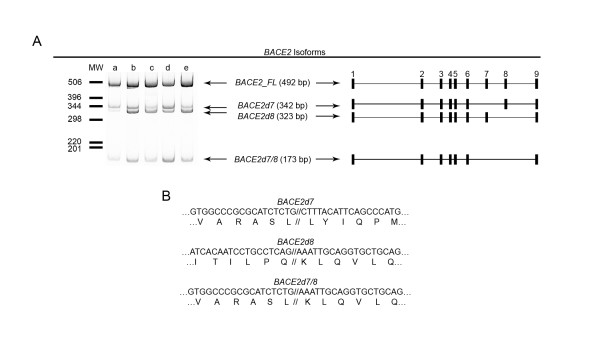
***BACE2 *isoforms present in human brain**. Human brain cDNA samples were screened for *BACE2 *isoforms using conventional PCR amplification across the alternatively spliced regions of exons 7 and 8. (A) Four alternatively spliced *BACE2 *isoforms were detected among multiple individuals. (B) Direct sequencing of the resulting splice variants confirmed their identities as *BACE2d7*, *BACE2d8 *and *BACE2d7/8*. Deletion of *BACE2 *exon 7 results in an in-frame deletion of 50 amino acids whereas deletions of *BACE2 *exon 8 or exons 7 and 8 lead to a frameshift and premature truncation of the protein.

**Figure 3:**
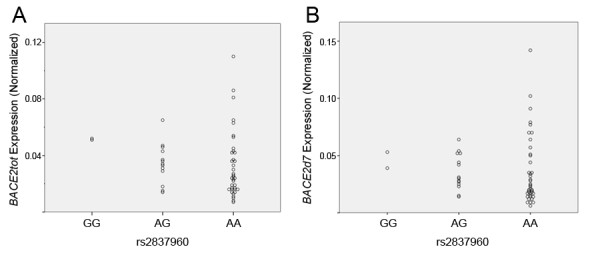
**Quantification of *BACE2tot *and *BACE2d7 *in human brain**. Real-time PCR with isoform-specific primers was used to quantify the expression of *BACE2tot *and *BACE2d7 *in cDNA prepared from human brain. (A-B) Samples exhibit a trend toward increased expression of both *BACE2tot *and *BACE2d7 *in the presence of rs2837960_G (p = 0.07 and p = 0.10, respectively, using Jonckheere-Terpstra testing).

To pursue this hypothesis, we sought to directly quantify *BACE2 *isoforms as a function of rs2837960 in a series of human brain samples. We began by confirming the presence of previously identified *BACE2 *isoforms that lack exons seven or eight (*BACE2d7 *and *BACE2d8*, respectively) as well as the full-length, nine-exon *BACE2 *transcript (*BACE2_FL*). We also identified a novel isoform lacking both exons 7 and 8 (*BACE2d7/8*) that was confirmed by direct sequencing (Figure 2). Considering the biological relevance of these isoforms, we note that the loss of exon 7 is predicted to result in an in-frame deletion of 50 amino acids, resulting in a 50.3 kDa peptide. Moreover, the BACE2 protein found in human brain appears to correspond to the *BACE2d7 *isoform based upon its size and pattern of epitope reactivity [14]. In contrast, the loss of exon 8 or exons 7-8 results in a frameshift and prematurely truncated BACE2 proteins of 43.0 kDa and 37.5 kDa, respectively. Hence, we chose to use real-time PCR to quantify *BACE2*tot and *BACE2d7 *since they represent the expression of total BACE2 and functional BACE2, respectively. A total of 53 brain cDNA samples were analyzed for genotypic associations between rs2837960 and expression of *BACE2tot *and *BACEd7*. This effort revealed a trend between rs2837960 and the expression of *BACE2tot *(p = 0.07) and *BACE2d7 *(p = 0.10, Figure 3). It is noteworthy that the trend toward greater expression of *BACE2tot *and *BACE2d7 *in rs2837960 minor allele carriers in our results is similar to the results from SNPExpress.

## Discussion

The primary finding of this investigation is that the majority of seventeen SNPs that exhibit a genome-wide significant association with RA are not associated with AD. Furthermore, the minor allele of rs2837960, which was found to be significantly associated with AD risk after combined analysis of Stage 1 and age-matched Stage 2 data, was associated with an increased risk of both RA and AD. Hence, these results contest the hypothesis that genetics underlie the inverse relationship between RA and AD, i.e. that alleles associated with an increased risk of RA are protective against AD. A secondary finding is that we have pursued the role of rs2837960 in its possible regulation of the nearby *BACE2 *gene. We report the presence of multiple *BACE2 *isoforms in human brain and that rs2837960 shows a trend for association with *BACE2*tot and *BACE2d7*, which represent total BACE2 and functional BACE2, respectively [14]. In summary, the genetic underpinnings of RA have negligible overlap with AD with the exception of rs2837960, which is associated with both RA and AD, possibly through its effects on *BACE2 *expression.

RA and AD each have a strong genetic component that accounts for approximately 50% and 60% of their risk, respectively [4,5]. The remainder of RA and AD risk is likely derived from environmental influences. The vast majority of RA-associated SNPs implicate gene products involved in immune system processes. Chronic inflammation of the brain is a common feature of AD pathology, raising the possibility that RA-associated SNPs that influence immune system function could influence AD risk [25-27]. It is well established that some of the most strongly AD-associated genes, including *CLU*, *CR1*, *TNF *and *CCR2*, exhibit ontological association with immune system processes [28-36]. Hence, the impetus for pursuing genetic overlap between RA and AD is greater than that provided by their epidemiologic relationship alone. However, our results indicate that RA-associated SNPs, which pertain largely to gene products involved in immune system processes, are not associated with AD.

There are several possible interpretations of our primary findings. The lack of overlap between RA-associated SNPs and AD could be due in part to the tissue-specific expression of DNA and RNA binding proteins required to interact with these SNPs to manifest effects on gene expression [37]. However, if any of the seventeen RA-associated SNPs included in this study are capable of modulating peripheral immune system activity, either alone or in combination with each other, then it is probable that their peripheral effects on the immune system would indirectly affect immune system activity within the CNS. Evidence supporting the ability of peripheral inflammation to modulate CNS inflammation has been reported previously [38]. Therefore, if RA-associated SNPs are only functional in the periphery then their effects on immune system function and inflammation should manifest in the CNS, even if the same SNPs do not modulate endogenous immune system function within the brain.

What is yet unclear is whether RA-associated alleles actually propagate inflammation and, if so, why they would not be expected to increase, rather than decrease, AD risk. In fact, the results of our study suggest that alleles that increase RA risk may likewise increase AD risk, i.e., rs2738960_G increases risk of both RA and AD, while rs3761847_G and rs2002842_A show a similar trend. If these observations are replicated in future studies, alleles that are pro-inflammatory may emerge as risk factors for both RA and AD. More explicitly, considering the role of genetics and environment in RA and AD, these results suggest that RA genetics alone may enhance rather than reduce AD risk. Hence, the inverse epidemiologic relationship between RA and AD is likely explained by an environmental RA-associated influence. In this regard, McGeer *et al. *postulated that the reduced prevalence of AD in RA patients is related to the use of anti-inflammatory drugs for the treatment of RA [2]. Multiple studies of anti-inflammatory agents have since been performed to test for their ability to modify AD risk and cognitive decline in AD patients, yielding mixed results [8,39-42]. To some extent, variability in study outcome may be explained by the additional ability of a subset of anti-inflammatory medications to reduce production of the neurotoxic Aβ_1-42 _peptide [6]. Further investigation is required to clarify the functional genetics of RA- and AD-associated SNPs and the role of anti-inflammatory medications in AD.

In pursuit of the functional genetics of rs2837960, which is associated with an increased risk of RA and AD, we investigated its association with the expression of *BACE2 *isoforms in human brain [43]. Thus, our secondary finding is that the minor allele of rs2837960 showed a strong trend for association with increased expression of *BACE2tot *and *BACE2d7*, the latter of which may represent the majority of functional BACE2 in human brain [14]. *BACE2 *encodes a transmembrane aspartic protease and is ~75% homologous with *BACE1 *with regard to amino acid sequence [20]. Although the function of BACE2 is disputed, it appears to possess both β-secretase and α-secretase-like activities [15]. Data obtained from the study of *BACE1/BACE2 *double-knockout mice suggest that *BACE2 *expressed in glia contributes significantly to Aβ production [44]. This glial-specific expression is likely due to the more distal of the two distinct *BACE2 *promoters, neither of which share similarity with the *BACE1 *promoter [18-20].

Several factors are consistent with the possibility that rs2837960, or SNPs in tight linkage with rs2837960 (LD of r^2 ^>0.8), are functional in modulating *BACE2 *expression. This evidence includes the observation that (i) rs2837960 resides within a haplotypic block that spans the region containing both the proximal and distal *BACE2 *promoters as well as the 5'UTR and first exon of *BACE2*, (ii) the region surrounding rs2837960 and its proxy SNPs (r^2 ^= 1.0, ~4 kb window) is well conserved in primates per rVISTA analysis (data not shown), and (iii) the alleles of rs2837960 and its proxy SNPs are predicted to differentially affect transcription factor binding per PROMO 3.0 analysis of the TRANSFAC database (data not shown) [45-47].

Other studies that examined the association between *BACE2 *polymorphisms and AD risk have yielded mixed results [48-54]. These studies differ with our study in that (i) they have focused on SNPs much more proximal to *BACE2 *that are not in strong linkage disequilibrium with rs2837960 and (ii) they generally utilized smaller populations than those utilized in our present study. Future analyses of the association between *BACE2 *SNPs and AD should therefore take into account SNPs that are more distal to *BACE2*, such as rs2837960, as well as utilize larger population sizes that are sufficiently powered to detect associations with AD. Thus, in future studies rs2837960 may emerge as a risk factor for both RA and AD that functionally modulates *BACE2 *expression. Elucidation of the precise mechanism by which rs2837960, or a SNP that is proxy to it, modulates *BACE2 *expression may contribute to a better understanding of the role of *BACE2 *in both AD and RA pathology.

## Conclusions

In summary, we have provided evidence that RA genetics do not underlie the inverse relationship between RA and AD but rather may promote AD. Furthermore, we have found that rs2837960 is associated with both RA and AD and that it, or one of its proxy SNPs, may modulate the expression of *BACE2*. As we learn more about the pathologic processes behind both RA and AD, including the contribution of *BACE2 *to each disease, a greater understanding of the factors underlying the inverse relationship between these two diseases may be obtained.

## Materials and methods

### SNP Selection

The Human Genome (HuGE) Navigator (http://www.hugenavigator.net) was queried using the search term "rheumatoid arthritis" to identify RA-associated SNPs of genome-wide significance [55]. Six available studies utilizing individuals of European decent were chosen to mimic the AD MAYO GWAS demographics (Table 7). Sample sizes ranged from ~1,600 (810 RA, 794 non-RA) to ~25,500 (7,322 RA, 18,262 non-RA). Thus, we identified twenty-eight candidate SNPs for study from the literature. SNPs which appeared more than once or that were in tight linkage disequilibrium with each other, i.e. r^2 ^>0.8 (according to the CEU HapMap population), were considered to be redundant and only those with the lowest RA-associated p-value were retained for further analysis [17]. This effort reduced the number of candidate SNPs to twenty-two. If a candidate RA-associated SNP was not available within the Mayo Clinic AD GWAS, an appropriate proxy SNP (LD of r^2 ^>0.8) was selected by using the HapMap-based SNAP proxy search (http://www.broadinstitute.org/mpg/snap/) [56]. Ultimately, seventeen of the candidate SNPs or their proxies were present in our AD GWAS dataset.

**Table 7:** RA GWAS reports identifying RA genetic risk factors

Article	PMID	# of GWAS Hits
**Gregersen et al., *Nat Genet*, 2009**	19503088	5
**Raychaudhuri et al., *Nat Genet*, 2008**	18794853	9
**Julia et al., *Arthritis Rheum*, 2008**	18668548	2
**WTCCC, *Nature*, 2007**	17554300	7
**Plenge et al., *N Engl J Med*, 2007**	17804836	3
**Plenge et al., *Nat Genet*, 2007**	17982456	2

Six RA GWAS manuscripts were identified by querying the HuGE Navigator database for "rheumatoid arthritis." Together, these studies document twenty-eight SNPs that are significantly associated with RA, p < 10^-6^. Of these twenty-eight SNPs, seventeen (or their proxies) were present in our AD GWAS and evaluated further for their association with AD.

### Case and Control Samples

The Mayo Clinic case-control samples used for the Stage 1 analysis have been described in detail in a prior GWAS publication [57]. The Mayo Clinic case-control series used for the Stage 2 study have also been previously described [58]. Briefly, clinical diagnoses of probable AD were made according to NINCDS-ADRDA criteria for samples from Jacksonville, FL (JS) and Rochester, MN (RS); age-matched controls had a score of 0 on the Clinical Dementia Rating scale. Additional samples were obtained from the Mayo Clinic brain bank (AUT); autopsy-confirmed diagnosis of AD (NINCDS-ADRDA, Braak score >4.0) was utilized for AD samples while non-AD samples exhibited limited AD pathology (Braak <2.5, not including other unrelated pathology).

### AD Association Testing

Association testing of RA-associated SNPs for AD risk was carried out in two stages by using PLINK software (http://pngu.mgh.harvard.edu/purcell/plink/) [59]. All genotyped samples were subject to strict quality control including elimination of samples with call rates <90%, MAF <0.01, HW p < 0.001, discrepancy between reported and genotyped sex, cryptic relatedness and discordant genotype clustering. Stage 1 consisted of 1264 non-AD and 843 AD subjects with average ages of 74.3 ± 4.5 (age at last assessment, mean ± SD) and 72.4 ± 4.6 years (age at diagnosis), respectively. The non-AD and AD groups in this series consisted of 51.7% and 57.5% female individuals, respectively. Stage 1 samples were genotyped by using HumanHap300-Duo Genotyping BeadChips processed with an Illumina BeadLab station (Illumina, San Diego, CA) at the Mayo Clinic Genotyping Shared Resource center (Rochester, MN).

We proceeded to test for an association between the seventeen RA-associated SNPs and AD in this Stage 1 case-control population. Stage 1 association testing was performed by using PLINK to generate allelic models that included odds ratios (OR), 95% confidence intervals (CI)s and uncorrected p-values. Logistic regression was also performed using the covariates age, sex and *APOE *genotype. With regards to multiple testing we expected to obtain approximately one false positive result given α = 0.05 (seventeen unique SNPs; 17 tests × 0.05 = 0.85). Bonferroni correction for multiple testing was also applied to data generated using allelic models.

Stage 2 samples were genotyped by using SEQUENOM MassARRAY iPLEX Platform (Sequenom, San Diego, CA). Overall, Stage 2 consisted of 2677 non-AD and 1102 AD subjects with average ages of 81.0 ± 6.2 and 83.5 ± 6.6 years of age, respectively. The non-AD and AD groups were composed of 55.0% and 64.0% female individuals, respectively. Stage 2 AD-SNP association testing was performed using only the three SNPs identified in Stage 1 as being associated with both RA and AD. PLINK software was used to generate odds ratios, 95% CIs and p-values per allelic modeling. Logistic regression including the covariates age, sex and *APOE *genotype was also performed. To evaluate the overall significance of Stage 1 and 2 data, they were combined and examined collectively.

Due to the considerable difference in mean age between Stage 1 and Stage 2 individuals, and due to our interest in focusing on genetic, rather than environmental factors, we also chose to examine only Stage 2 individuals between 60 and 80 years of age. Hence, when Stage 2 was limited to individuals between 60 and 80 years of age, our analysis included 912 non-AD and 186 AD subjects with average ages of 73.9 ± 3.8 and 72.8 ± 5.1 years. The non-AD and AD groups consisted of 49.9% and 57.0% female individuals, respectively. Similar to our analysis of our initial Stage 2 population, logistic regression of this modified Stage 2 population was also performed to test for an association between the three AD-associated SNPs from Stage 1. Furthermore, we evaluated the overall significance of RA-associated SNP associations with AD in combined Stage 1 and Stage 2 individuals between 60 and 80 years of age.

### Human Tissue

Human anterior cingulate brain specimens were generously provided by the Sanders-Brown AD Center Neuropathology Core and have been described elsewhere [60]. The samples were from deceased individuals with an average age at death for females of 82 ± 7 years (mean ± SD, n = 29) and for males of 81 ± 8 (n = 24). The average postmortem interval (PMI) for females and males was 3.2 ± 0.8 h and 3.0 ± 0.8 h, respectively.

### Evaluation of BACE2 isoforms in vivo

To gain insights into the functionality of rs2837960 we tested for an association between rs2837960 and *BACE2 *expression in human brain. We first screened anterior cingulate samples for the presence of *BACE2 *and its known alternatively spliced isoforms that lack exons 7 and 8, respectively. Total RNA and genomic DNA were prepared from human tissue samples; the RNA was reverse transcribed as we have reported elsewhere [61,62]. Conventional PCR using Platinum Taq (Invitrogen, Carlsbad, CA) was used to amplify the region of *BACE2 *spanning exons 6-9 (Table 8). Thermal cycling conditions consisted of denaturation at 95°C for 5 min followed by 32 cycles of 95°C for 30 s, 60°C for 30 s, 72°C for 1 min and a final extension at 72°C for 2 min. PCR products were separated using 8% TBE-PAGE gel electrophoresis and visualized using SYBR-gold fluorescent stain (Invitrogen) and a fluorescence imager (FLA-2000, Fuji). To confirm the identities of the *BACE2 *splice variants, bands were excised, purified and directly sequenced (Davis Sequencing, Davis, CA).

**Table 8:** Primers used for analyses of *BACE2 *isoform expression.

*BACE2 *Isoform screening primers	
*BACE2 *Exon 6 Forward:	ATAACGCAGACAAGGCCATC

*BACE2 *Exon 9 Reverse:	GGACACAGTTGCTGGCTACA

***BACE2 *isoform specific RT-PCR primers**	

*BACE2 *Exon 6 Forward:	GCCCCAGAAGGTGTTTGAT

*BACE2 *Exon 6-8 Junction Reverse:	GGCTGAATGTAAAGCAGAG

*BACE2 *Exon 5 Forward:	TGGGTGGAATTGAACCAAGT

*BACE2 *Exon 6 Reverse:	GATGGCCTTGTCTGCGTTAT

To quantify total *BACE2 *expression (*BACE2tot*) and expression of the *BACE2 *isoform lacking exon 7 (*BACE2d7*) we designed separate primer sets. *BACE2tot *expression was measured by amplification of a product spanning a non-alternatively spliced region of *BACE2 *(exons 5-6). Isoform-specific primers designed to amplify *BACE2d7 *consisted of a forward primer specific to *BACE2 *exon 6 and a reverse primer specific to the junction of exons 6-8 (Table 8). DNA samples were genotyped using a TaqMan SNP Genotyping Assay (ID # C_2688271_10; ABI, Carlsbad, CA).

Quantitative real-time PCR reactions contained ~20 ng of sample cDNA together with 10 μl of PerfeCTA SYBR green SuperMix (Quanta Biosciences, Gaithersburg, MD), 10 μl of ddH2O and 20 pmol of forward and reverse primers. Cycling conditions included a 3 minute denaturation step at 95°C followed by 40 cycles of denaturation for 15 seconds at 95°C and annealing/extension for 45 seconds at 60°C using an MJ Opticon 4 thermal cycler (Biorad, Hercules, CA). A melting curve was generated following cycling to assess the purity of amplification product. Fidelity of amplification was also assessed via visual inspection of PCR products on 8% TBE-PAGE gel stained with SYBR gold. Standard curves were generated from purified PCR products that were quantified by A260/280 spectrophotometric analysis. Standard curves were then used to calculate the copy number for each *BACE2 *isoform measured.

Hypoxanthine-guanine phosphoribosyltransferase (*HPRT*) and ribosomal protein L32 (*RPL32*) were used as housekeeping genes per the analysis of geNorm software as described previously [63-65]. Expression levels of each of these genes were measured by using quantitative real-time PCR and gene specific primers under conditions identical to cycling conditions for *BACE2*. Standard curves were used to generate exact copy numbers, which in turn were used to calculate the sample-specific geometric mean of *HPRT *and *RPL32 *expression. The geometric mean was in turn used to normalize subsequent *BACE2 *expression data. Analysis of the association between *BACE2 *isoforms and rs2837960 genotype was performed using non-parametric Jonckheere-Terpstra testing (PASW Statistics, v.18, IBM, Somers, NY).

## Competing interests

The authors declare that they have no competing interests.

## Authors' contributions

CRS and SE contributed to experimental design and wrote the manuscript. CRS performed RT-PCR of *BACE2 *isoforms, rs2837960 genotyping of human brain tissue and association testing between *BACE2 *isoform expression and rs2837960. FZ performed SEQUENOM genotyping of samples for Stage 2 analyses. SGY, FZ and CRS performed statistical analysis of RA-associated SNPs and AD GWAS data. All authors have read and approved the final manuscript.
